# Hospice Readmission, Hospitalization, and Hospital Death Among Patients Discharged Alive from Hospice

**DOI:** 10.1001/jamanetworkopen.2024.11520

**Published:** 2024-05-16

**Authors:** Elizabeth A. Luth, Caitlin Brennan, Susan L. Hurley, Veerawat Phongtankuel, Holly G. Prigerson, Miriam Ryvicker, Hui Shao, Yongkang Zhang

**Affiliations:** 1 Rutgers University, New Brunswick, New Jersey; 2 Care Dimensions, Danvers, Massachusetts; 3 Weill Cornell Medicine, New York, New York; 4 VNS Health, New York, New York; 5 Emory University, Gainesville, Georgia

## Abstract

**Importance:**

Transitions in care settings following live discharge from hospice care are burdensome for patients and families. Factors contributing to risk of burdensome transitions following hospice discharge are understudied.

**Objective:**

To identify factors associated with 2 burdensome transitions following hospice live discharge, as defined by the Centers for Medicare & Medicaid Services.

**Design, Setting, and Participants:**

This population-based retrospective cohort study included a 20% random sample of Medicare fee-for-service beneficiaries using 2014 to 2019 Medicare claims data. Data were analyzed from April 22, 2023, to March 4, 2024.

**Exposure:**

Live hospice discharge.

**Main Outcomes and Measures:**

Multivariable logistic regression examined associations among patient, health care provision, and organizational characteristics with 2 burdensome transitions after live hospice discharge (outcomes): type 1, hospice discharge, hospitalization within 2 days, and hospice readmission within 2 days; and type 2, hospice discharge, hospitalization within 2 days, and hospital death.

**Results:**

This study included 115 072 Medicare beneficiaries discharged alive from hospice (mean [SD] age, 84.4 [6.6] years; 71892 [62.5%] female; 5462 [4.8%] Hispanic, 9822 [8.5%] non-Hispanic Black, and 96 115 [83.5%] non-Hispanic White). Overall, 10 381 individuals (9.0%) experienced a type 1 burdensome transition and 3144 individuals (2.7%) experienced a type 2 burdensome transition. In adjusted models, factors associated with higher odds of burdensome transitions included identifying as non-Hispanic Black (type 1: adjusted odds ratio [aOR], 1.47; 95% CI, 1.36-1.58; type 2: aOR, 1.70; 95% CI, 1.51-1.90), hospice stays of 7 days or fewer (type 1: aOR, 1.13; 95% CI, 1.06-1.21; type 2: aOR, 1.71; 95% CI, 1.53-1.90), and care from a for-profit hospice (type 1: aOR, 1.78; 95% CI, 1.62-1.96; type 2: aOR, 1.32; 95% CI, 1.15-1.52). Nursing home residence (type 1: aOR, 0.66; 95% CI, 0.61-0.72; type 2: aOR, 0.47; 95% CI, 0.40-0.54) and hospice stays of 180 days or longer (type 1: aOR, 0.63; 95% CI, 0.59-0.68; type 2: aOR, 0.60; 95% CI, 0.52-0.69) were associated with lower odds of burdensome transitions.

**Conclusion and Relevance:**

This retrospective cohort study of burdensome transitions following live hospice discharge found that non-Hispanic Black race, short hospice stays, and care from for-profit hospices were associated with higher odds of experiencing a burdensome transition. These findings suggest that changes to clinical practice and policy may reduce the risk of burdensome transitions, such as hospice discharge planning that is incentivized, systematically applied, and tailored to needs of patients at greater risk for burdensome transitions.

## Key Points

**Question:**

What factors are associated with burdensome transitions 2 days after live hospice discharge?

**Findings:**

This cohort study of 115 072 Medicare fee-for-service beneficiaries from 2014 to 2019 found that 9% of individuals discharged alive from hospice were hospitalized and readmitted to hospice and 3% were hospitalized and died in the hospital. Identifying as Black, having a short hospice stay, and receiving care from a for-profit hospice were associated with higher odds of burdensome transition.

**Meaning:**

These findings suggest that clinical practice and policy should attend to patients at greater risk for burdensome transitions after hospice live discharge, including systematic, incentivized discharge planning tailored to individual patient needs.

## Teaser

This cohort study assesses patient, health care, and organizational factors associated with hospice readmission, hospitalization, and in-hospital mortality among Medicare fee-for-service beneficiaries discharged alive from hospice.

## Introduction

Live discharge from hospice—experienced by 15% of Medicare hospice users in 2020^1^—occurs when an individual leaves hospice before death. Reasons for live discharge include unplanned hospitalization, seeking curative treatment for a terminal condition, transferring hospice services, or condition stabilization that makes someone ineligible for hospice. Live discharge has policy, patient, and caregiver consequences.^2,3,4^ It is typically disruptive, resulting in the loss of clinical and support services during the critical end-of-life period.^2,3,4^ Nearly half of hospice patients (42%) die within 6 months of live discharge,^5^ suggesting that uninterrupted hospice care may be appropriate for many individuals who were discharged alive.

The Centers for Medicare & Medicaid Services (CMS) are concerned about the number of hospice live discharges and potentially negative consequences for patient quality of life and death. In 2021, CMS added 4 measures related to hospice live discharge to their 10-item Hospice Care Index for hospice care quality.^6^ These 4 measures include early (ie, ≤7 days of hospice enrollment) and late (ie, >180 days of hospice enrollment) live discharges and 2 types of posthospice burdensome discharge transition experiences.^6^ Type 1 burdensome transitions focus on individuals who are admitted to a hospital within 2 days following hospice live discharge, and then readmitted to hospice within 2 days of hospital discharge.^6^ Type 2 burdensome transitions identifies individuals who are hospitalized within 2 days after hospice live discharge and die while hospitalized.^6^ Early and late live discharges are associated with racial and ethnic minoritized status,^7,8,9,10,11^ younger age,^7,8,12,13^ dual Medicare and Medicaid enrollment,^7,8,9^ fewer comorbidities,^7,14^ increased functional status,^7,15^ and for-profit hospice status.^10,16^ However, type 1 and 2 burdensome transitions have not been as well studied (a 2016 study by Prsic et al^16^ is an exception), despite being potentially related to poor assessment of patient stability prior to live discharge^6^ or nonsystematic approaches to live discharge planning^17,18^ that may result in postdischarge care fragmentation.^19^

In this study, we address the question of what individual patient, health care provision, and hospice organizational factors are associated with the 2 types of burdensome transitions following live discharge from hospice, as defined by CMS. Identifying factors associated with burdensome transitions is a necessary first step in identifying immediate and longer-term targets for intervention to improve end-of-life outcomes. Drawing from prior literature and a modified Holzemer framework,^20^ we selected factors associated with increased risk of live discharge (eg, racial and ethnic minoritized status, dual enrollment, for-profit status) and factors believed to be associated with protection against live discharge (eg, older age, fewer comorbidities, less frailty, palliative care consultation). Patient sociodemographic and health characteristics could help identify which patients may require additional attention during live discharge if they have increased risk of postdischarge burdensome transitions. Aspects of hospice care are potentially modifiable in current clinical practice and future policies. Health care provision also includes ongoing processes, such as goals of care planning, that may impact postdischarge care trajectories. Organizational factors related to the hospice care setting could help identify potential targets for regulatory oversight.

## Methods

This cohort study was approved by the Institutional Review Board at Weill Cornell Medicine with a waiver of consent because this study uses deidentified secondary claims data collected by CMS. We followed the Strengthening the Reporting of Observational Studies in Epidemiology (STROBE) reporting guideline.

### Data Sources and Study Sample

We conducted a retrospective cohort study using a 20% random sample of 2014 to 2019 Medicare fee-for-service (FFS) beneficiaries. Medicare is the federally funded health insurance program in the US for individuals aged 65 years and older and for eligible individuals with end-stage kidney disease and disabilities.^21,22,23^ We used Medicare hospice claims files to identify hospice live discharges using discharge status codes.^1,24^ To exclude most hospice stays that might be readmissions following a hospice discharge in 2013, we implemented a washout period of the first 90 days of 2014 to only include patients who newly started their hospice benefits in the study period.^25^ The analysis included 115 072 patients who were aged 65 years or older when admitted to hospice, continuously enrolled in Medicare Parts A and B for 12 months before hospice admission, and continuously enrolled in Medicare Parts A and B after hospice discharge until death or the end of the study period (December 31, 2019). In rare cases, if a patient had more than 1 hospice live discharge, we analyzed the first one.

We used Master Beneficiary Summary Files to extract patient demographics and enrollment information. We used hospice claims to identify service location, hospice services provided, principal diagnosis, and other hospice stay characteristics. We used Medicare Parts A (eg, inpatient hospital, skilled nursing facility, and home health^21^) and B (eg, physician and outpatient services^21^) files to identify claims related to hospital stays, patient health status, services prior to hospice admission, and burdensome transitions after hospice discharge. We extracted hospice ownership from CMS Provider of Services files^26^ and hospice size data from CMS Medicare Post-Acute Care & Hospice^27^ utilization and payment data. There were no missing data on any variables used for analysis.

### Burdensome Transition Measures

We calculated 2 burdensome transition measures, as defined by CMS as part of the Hospice Care Index. Type 1 was defined as hospitalization within 2 days after hospice live discharge, followed by hospice readmission within 2 days of hospital discharge. Type 2 was defined as hospitalization within 2 days after hospice live discharge with in-hospital death.^24^

### Individual Patient, Health Care Provision, and Organizational Setting Characteristics

We examined individual patient (sociodemographic and health), health care provision, and organizational characteristics that might be associated with burdensome transitions after hospice live discharge. Patient sociodemographic characteristics included age at hospice admission (65-74, 75-84, ≥85 years), sex (male, female), race and ethnicity as recorded in the Master Beneficiary Summary Files^28^ (categorized as Hispanic, non-Hispanic Black, non-Hispanic White, and other [including American Indian or Alaska Native, Asian or Pacific Islander, or unknown]), dual enrollment status (Medicare-Medicaid, Medicare only), residence before hospice admission (urban/suburban, rural by zip code),^29^ and long-term nursing home residence prior to hospice admission.^30^ We used a categorical claims-based frailty index (not frail, prefrail, mildly frail, moderately to severely frail),^31,32,33^ CMS Hierarchical Condition Category (HCC) score as a continuous variable, and principal diagnosis, including Alzheimer Disease and related dementias, the 5 most common cancers among Medicare beneficiaries (defined by the Chronic Conditions Data Warehouse: breast, colorectal, endometrial, lung, or prostate), cardiovascular disease (eg, heart failure, acute myocardial infarction, and ischemic heart disease), chronic kidney disease, and COPD (as these are the most common diagnoses among hospice patients),^34^ or other to measure patient health status.

We examined several health care characteristics. The Medicare hospice benefit covers 4 levels of hospice care depending on patient and caregiver needs.^35^ Routine home care, accounting for 99% of hospice days,^1^ provides comfort and symptom management. It is delivered in the community or patient residence, assisted living, nursing home, inpatient hospital, hospice residence, or other settings. We measured location of hospice care as the location where most routine home care was delivered. Patients and caregivers can receive care in addition to routine home care, which may signal severe patient conditions. Therefore, we identified the use of continuous home care (management of acute medical symptoms, such as uncontrolled pain), inpatient respite care (short-term relief for family caregivers), and general inpatient care (GIP; short-term hospital care for symptom management) separately as dichotomous variables (yes or no). We also measured length of stay (short, ≤7 days; expected, 8-179 days; long, ≥180 days), and live discharge reason. Live discharge reasons included discharge home with cause, patient revocation of hospice benefits (eg, seeking curative treatment for a terminal condition), condition stabilization, patient unavailability, unplanned hospitalization, or transfer to another hospice. Goals of care planning were assessed using proxy measures, including advance care planning before hospice discharge (starting in 2016) (Current Procedural Terminology codes 99497 and 99498) and any palliative care encounter 6 months before hospice admission (*International Statistical Classification of Diseases and Related Health Problems, Tenth Revision *[*ICD-10*] code Z51.1).

For organizational setting characteristics, we examined hospice ownership (nonprofit, for-profit, government, other) and size (quintiles of number of total stays in each calendar year). We included hospice admission and discharge years as controls.

### Statistical Analysis

We compared individual, health care provision, and organizational factors by burdensome transition outcome status using χ^2^ tests for categorical variables and *t* tests for continuous variables. For each burdensome transition outcome, we used multivariable logistic regression to examine factors associated with the likelihood of the outcome vs no burdensome transition. Individual, care, and organizational factors and admission year and discharge year fixed effects were included in the model. We used robust standard errors clustered by hospice organization to account for correlations of patients discharged from the same hospice.

In secondary analysis, we mapped patient residential zip codes into hospital referral regions (HRRs) and included them as fixed effects to account for regional variations in end-of-life health care preferences and hospice market characteristics. To facilitate comparisons across different categories of key factors associated with burdensome transitions (race and ethnicity, frailty, location and type of hospice care, hospice ownership), we calculated estimated probabilities, holding all other variables constant at their means.

*P* values were 2-sided, and statistical significance was set at *P* < .05. Analyses were conducted using Stata version 17 (StataCorp) from April 22, 2023, to March 4, 2024.

## Results

### Descriptive Statistics

This study included 115 072 Medicare FFS beneficiaries discharged alive from hospice (mean [SD] age, 84.4 [6.6] years; 71 892 [62.5%] female; 5462 Hispanic individuals [4.8%], 9822 non-Hispanic Black individuals [8.5%], and 96 115 non-Hispanic White individuals [83.5%]). Burdensome transitions accounted for 11.7% of live discharges: 10 381 individuals (9.0%) experienced a type 1 transition (live discharge, hospitalization within 2 days, hospice readmission within 2 days) and 3144 individuals (2.7%) experienced a type 2 transition (live discharge, hospitalization within 2 days, hospital death). Individuals who experienced type 1 and 2 transitions differed from the general live discharge population for all sociodemographic, health, and hospice characteristics (Table 1).

**Table 1.** Descriptive Statistics for Selected Sample of Fee-for-Service Medicare Beneficiaries Discharged Alive From Hospice Care by Postdischarge Transition Type, 2014-2019^a^

Characteristic	Individuals, No. (%)			
	Total sample (N = 115 072)	No burdensome transition (n = 101 547)	Type 1 transition (n = 10 381)^b^	Type 2 transition (n = 3144)^c^
**Patient sociodemographic characteristics**				
Age, y				
65-74^d^	18 350 (15.9)	15 462 (15.2)	2123 (20.5)	765 (24.3)
75-84	35 497 (30.8)	30 836 (30.4)	3579 (34.5)	1082 (34.4)
≥85	61 225 (53.2)	55 249 (54.4)	4679 (45.1)	1297 (41.3)
Race and ethnicity				
Hispanic	5462 (4.7)	4696 (4.6)	567 (5.5)	199 (6.3)
Non-Hispanic Black	9822 (8.5)	8031 (7.9)	1321 (12.7)	470 (14.9)
Non-Hispanic White	96 115 (83.5)	85 595 (84.3)	8197 (79.0)	2323 (73.9)
Other^e^	3673 (3.2)	3225 (3.2)	296 (2.9)	152 (4.8)
Sex				
Male^d^	43 180 (37.5)	37 305 (36.7)	4414 (42.5)	1461 (46.5)
Female	71 892 (62.5)	64 242 (63.3)	5967 (57.5)	1683 (53.5)
Medicare and/or Medicaid enrollment				
Medicare only^d^	77 791 (67.6)	68 321 (67.3)	7359 (70.9)	2111 (67.1)
Dual Medicare and Medicaid	37 281 (32.4)	33 226 (32.7)	3022 (29.1)	1033 (32.9)
Residence				
Urban or suburban	97 939 (85.1)	86 240 (84.9)	9034 (87.0)	2665 (84.8)
Rural	17 133 (14.9)	15 307 (15.1)	1347 (13.0)	479 (15.2)
Residence prior to admission				
Community^d^	91 344 (79.4)	79 443 (78.2)	9092 (87.6)	2809 (89.3)
Nursing home	23 728 (20.6)	22 104 (21.8)	1289 (12.4)	335 (10.7)
**Patient health characteristics**				
Frailty Index				
Not frail^d^	8087 (7.0)	7318 (7.2)	558 (5.4)	211 (6.7)
Prefrail	38 189 (33.2)	33 682 (33.2)	3412 (32.9)	1095 (34.8)
Mildly frail	51 925 (45.1)	45 910 (45.2)	4623 (44.5)	1392 (44.3)
Moderately to severely frail	16 871 (14.7)	14 637 (14.4)	1788 (17.2)	446 (14.2)
CMS HCC Score, mean (SD)	2.44 (1.7)	2.38 (1.6)	2.85 (1.9)	2.98 (2.0)
Principal diagnosis				
ADRD	21 004 (18.3)	18 745 (18.5)	1780 (17.1)	479 (15.2)
Cancer	9688 (18.3)	7981 (7.9)	1291 (11.7)	488 (15.5)
Cardiovascular disease	18 937 (16.5)	16 599 (16.3)	1835 (17.7)	543 (17.3)
Chronic kidney disease	4150 (3.6)	3609 (3.6)	387 (3.7)	154 (4.9)
COPD	10 495 (9.1)	8901 (8.8)	1211 (11.7)	383 (12.2)
Other^d^	50 798 (44.1)	45 752 (45.1)	3949 (39.0)	1097 (34.9)
**Health care provision**				
Place hospice was received				
Community^d^	67 507 (58.7)	58 281 (57.4)	7034 (67.8)	2192 (69.7)
Assisted living	17 946 (15.6)	16 155 (15.9)	1536 (14.8)	255 (8.1)
Nursing home	23 836 (20.7)	21 970 (21.6)	1411 (13.6)	455 (14.5)
Inpatient hospital	2379 (2.1)	2011 (2.0)	200 (1.9)	168 (5.3)
Hospice residence	1849 (1.6)	1712 (1.7)	131 (1.3)	51 (1.6)
Other	1510 (1.3)	1418 (1.4)	69 (0.7)	23 (0.7)
Type of hospice care				
Continuous home care				
Yes	1446 (1.3)	1183 (1.2)	212 (2.0)	51 (1.6)
No^d^	113 626 (98.7)	100 364 (98.8)	10 169 (98.0)	3093 (98.4)
Inpatient respite				
Yes	5508 (4.8)	5017 (4.9)	373 (3.6)	118 (3.8)
No^d^	109 564 (95.2)	96 530 (95.1)	10 008 (96.4)	3026 (96.2)
General inpatient care				
Yes	8523 (7.4)	7572 (7.5)	638 (6.1)	313 (10.0)
No^d^	106 549 (92.6)	93 975 (92.5)	9743 (93.9)	2831 (90.0)
Length of stay, d				
≤7	11 816 (10.3)	9523 (9.4)	1554 (15.0)	739 (23.5)
8-179^d^	70 718 (61.5)	61 399 (60.5)	7280 (70.1)	2039 (64.9)
≥180	32 538 (28.3)	30 625 (30.2)	1547 (14.9)	366 (11.6)
Discharge reason				
Condition stabilization^d^	47 821 (41.6)	45 846 (45.1)	1553 (15.0)	422 (13.4)
With cause	1655 (1.4)	1490 (1.5)	127 (1.2)	38 (1.2)
Patient unavailability	8113 (7.1)	6233 (6.1)	1469 (14.2)	411 (13.1)
Revocation	48 320 (42.0)	40 649 (40.0)	5872 (56.6)	1799 (57.2)
Transfer to inpatient care	4570 (4.0)	2953 (2.9)	1218 (11.7)	399 (12.7)
Transfer to other facilities	4593 (4.0)	4376 (4.3)	142 (1.4)	75 (2.4)
ACP before hospice discharge				
Yes	10 973 (9.5)	9533 (9.4)	1110 (10.7)	330 (10.5)
No	104 099 (90.5)	92 014 (90.6)	9271 (89.3)	2814 (89.5)
Palliative care in 6 mo before hospice admission				
Yes	18 965 (16.5)	16 401 (16.2)	1862 (17.9)	702 (22.3)
No	96 107 (83.5)	85 146 (83.8)	8519 (82.1)	2442 (77.7)
**Organizational setting characteristics**				
Hospice size, quintile				
1 (Smallest)^d^	7220 (6.3)	6461 (6.4)	544 (5.2)	215 (6.8)
2	10 329 (9.0)	9161 (9.0)	883 (8.5)	285 (9.1)
3	14 598 (12.7)	12 869 (12.7)	1327 (12.8)	402 (12.8)
4	22 603 (19.6)	19 741 (19.4)	2195 (21.1)	667 (21.2)
5 (Largest)	60 322 (52.4)	53 315 (52.5)	5432 (52.3)	1575 (50.1)
Hospice ownership status				
Not-for-profit ^d^	39 513 (34.3)	36 094 (35.5)	2486 (23.9)	933 (32.8)
For-profit	61 446 (53.4)	52 846 (52.0)	6785 (65.4)	1815 (63.9)
Government	2001 (1.7)	1841 (1.8)	103 (1.0)	57 (2.0)
Other	12 112 (10.5)	10 766 (10.6)	1007 (9.7)	37 (1.3)

Abbreviations: ACP, advance care planning; ADRD, Alzheimer disease and related dementias; CMS, Centers for Medicare & Medicaid Services; COPD, chronic obstructive pulmonary disease; HCC, Hierarchical Condition Category.

^a^ All *P* < .001 when comparing no burdensome transition, Type 1, and Type 2 transitions using *t* tests for continuous variables and χ^2^ tests for categorical variables.

^b^ Type 1 transitions: hospitalization within 2 days of hospice live discharge followed by hospice readmission within 2 days of hospital discharge.

^c^ Type 2 transitions: hospitalization within 2 days of hospice live discharge followed by in-hospital death.

^d^ Reference group.

^e^ Includes American Indian or Alaska Native, Asian or Pacific Islander, or unknown.

### Factors Associated With Type 1 Transitions

Factors associated with lower odds of experiencing a live discharge followed by hospitalization and subsequent hospice readmission included female sex (adjusted odds ratio [aOR], 0.95; 95% CI, 0.91-0.99; *P* = .01) dual Medicare and Medicaid enrollment (aOR, 0.91; 95% CI, 0.86-0.96; *P* = .001), nursing home residence (aOR, 0.66; 95% CI, 0.61-0.72; *P* < .001), hospice stay of 180 days or longer (aOR, 0.63; 95% CI, 0.59-0.68, *P* < .001), and receiving inpatient respite (aOR, 0.78; 95% CI, 0.70-0.87; *P* < .001) or GIP (aOR, 0.85; 95% CI, 0.75-0.97; *P* = .01) care. Factors associated with greater odds of experiencing a type 1 burdensome transition included identifying as non-Hispanic Black (aOR, 1.47; 95% CI, 1.36-1.58; *P* < .001), having any degree of frailty (eg, prefrail: aOR, 1.37; 95% CI, 1.24-1.52; *P* < .001), a cancer (aOR, 1.18; 95% CI, 1.10-1.27; *P* < .001) or COPD (aOR, 1.18; 95% CI, 1.10-1.26; *P* < .001) diagnosis, higher HCC score (aOR, 1.07; 95% CI, 1.05-1.08; *P* < .001), short hospice stay (aOR, 1.13; 95% CI, 1.06-1.21; *P* < .001), palliative care consultation (aOR, 1.08; 95% CI, 1.01-1.14; *P* = .02), and receiving care from a larger (eg, quintile 5: aOR, 1.36; 95% CI, 1.21-1.52; *P* < .001) or for-profit (aOR, 1.78; 95% CI, 1.62-1.96; *P* < .001) hospice (Table 2).

**Table 2.** Logistic Regression Analysis to Identify Factors Associated With Burdensome Transition Type 1 in Hospice Patients Discharged Alive^a^

Factor	aOR (95% CI)	*P* value
**Patient sociodemographic characteristics**		
Age, y		
65-74	1 [Reference]	NA
75-84	0.96 (0.90-1.02)	.20
≥85	0.86 (0.81-0.92)	<.001
Race and ethnicity		
Hispanic	1.06 (0.93-1.20)	.37
Non-Hispanic Black	1.47 (1.36-1.58)	<.001
Non-Hispanic White	1 [Reference]	NA
Other^b^	0.89 (0.78-1.01)	.06
Sex		
Male	1 [Reference]	NA
Female	0.95 (0.91-0.99)	.01
Medicare and/or Medicaid enrollment status		
Medicare only	1 [Reference]	NA
Dual Medicare and Medicaid	0.91 (0.86-0.96)	.001
Residence		
Urban or suburban	1 [Reference]	NA
Rural	0.95 (0.87-1.04)	.25
Residence prior to hospice admission		
Community	1 [Reference]	NA
Nursing home	0.66 (0.61-0.72)	<.001
**Patient health characteristics**		
Frailty Index		
Not frail	1 [Reference]	NA
Prefrail	1.37 (1.24-1.52)	<.001
Mildly frail	1.49 (1.35-1.64)	<.001
Moderately to severely frail	1.78 (1.59-1.98)	<.001
CMS HCC score	1.07 (1.05-1.08)	<.001
Principal diagnosis		
ADRD	0.99 (0.94-1.06)	.93
Cancer	1.18 (1.10-1.27)	<.001
Cardiovascular disease	1.04 (0.98-1.11)	.17
Chronic kidney disease	0.95 (0.95-1.06)	.33
COPD	1.18 (1.10-1.26)	<.001
Other	1 [Reference]	NA
**Health care provision**		
Place hospice was received		
Community	1 [Reference]	NA
Assisted living	0.99 (0.92-1.05)	.69
Nursing home	0.87 (0.79-0.95)	.002
Inpatient hospital	0.75 (0.61-0.92)	.006
Hospice residence	0.68 (0.54-0.86)	.001
Other	0.52 (0.40-0.66)	<.001
Type of hospice care		
Continuous home care	1.47 (1.21-1.78)	<.001
Inpatient respite	0.78 (0.70-0.87)	<.001
General inpatient care	0.85 (0.75-0.97)	.01
Length of stay, d		
≤7	1.13 (1.06-1.21)	<.001
8-179	1 [Reference]	NA
≥180	0.63 (0.58-0.68)	<.001
Discharge reason		
Condition stabilization	1 [Reference]	NA
With cause	2.13 (1.71-2.65)	<.001
Patient unavailability	5.29 (4.60-6.08)	<.001
Revocation	3.44 (3.09-3.84)	<.001
Transfer to inpatient care	9.59 (8.35-11.01)	<.001
Transfer to other facilities	1.07 (0.83-1.37)	.61
ACP before hospice discharge	0.99 (0.93-1.07)	.97
Palliative care in 6 mo before hospice admission	1.08 (1.01-1.14)	.02
**Organizational setting characteristics**		
Hospice size, quintile		
1 (Smallest)	1 [Reference]	NA
2	1.18 (1.05-1.33)	.006
3	1.30 (1.16-1.45)	<.001
4	1.45 (1.30-1.63)	<.001
5 (Largest)	1.36 (1.21-1.52)	<.001
Hospice ownership status		
Not-for-profit	1 [Reference]	NA
For-profit	1.78 (1.62-1.96)	<.001
Government	1.05 (0.80-1.39)	.73
Other	1.29 (1.13-1.48)	<.001

Abbreviations: ACP, advance care planning; ADRD, Alzheimer disease and related dementias; aOR, adjusted odds ratios; CMS, Centers for Medicare & Medicaid Services; COPD, chronic obstructive pulmonary disease; HCC Score, Hierarchical Condition Category; NA, not applicable.

^a^ Type 1 transitions were hospitalization within 2 days of hospice live discharge followed by hospice readmission within 2 days of hospital discharge. Analysis also adjusts for hospice admission and discharge years.

^b^ Includes American Indian or Alaska Native, Asian or Pacific Islander, or unknown.

### Factors Associated With Type 2 Transitions

Factors associated with lower odds of experiencing a type 2 burdensome transition included older age (eg, ≥85 years: aOR, 0.83; 95% CI, 0.75-0.92; *P* < .001), female sex (aOR, 0.86; 95% CI, 0.80-0.93; *P* < .001), long hospice stay (aOR, 0.60; 95% CI, 0.52-0.69; *P* < .001), nursing home residence (aOR, 0.47; 95% CI, 0.40-0.54; *P* < .001), and receiving hospice in assisted living (aOR, 0.67; 95% CI, 0.59-0.77; *P* < .001) or a hospice residence (aOR, 0.63; 95% CI, 0.45-0.88; *P* < .007). Factors associated with greater odds of experiencing a type 2 burdensome transition included identifying as any race or ethnicity but non-Hispanic White (Hispanic: aOR, 1.23; 95% CI, 1.05-1.45; *P* = .01; non-Hispanic Black: aOR, 1.70; 95% CI, 1.51-1.90; *P* < .001), a cancer (aOR, 1.45; 95% CI, 1.29-1.62; *P* < .001) or COPD (aOR, 1.30; 95% CI, 1.13-1.49; *P* < .001) diagnosis, higher HCC score (aOR, 1.09; 95% CI, 1.07-1.12; *P* < .001), short hospice stay (aOR, 1.71; 95% CI, 1.53-1.90; *P* < .001), palliative care consultation (aOR, 1.27; 95% CI, 1.15-1.39; *P* < .001), receiving care from a government hospice (aOR, 1.38; 95% CI, 1.01-1.87; *P* = .04), and receiving care in a nursing home (aOR, 1.16; 95% CI, 1.02-1.33; *P* = .03) or inpatient hospital (aOR, 1.49; 95% CI, 1.12-1.98; *P* = .007) (Table 3).

**Table 3.** Logistic Regression Analysis to Identify Factors Associated With Burdensome Transition Type 2 in Hospice Patients Discharged Alive^a^

Factor	aOR (95% CI)	*P *value
**Patient sociodemographic characteristics**		
Age, y		
65-74	1 [Reference]	NA
75-84	0.90 (0.82-0.99)	.03
≥85	0.83 (0.75-0.92)	<.001
Race and ethnicity		
Hispanic	1.23 (1.05-1.45)	.01
Non-Hispanic Black	1.70 (1.51-1.90)	<.001
Non-Hispanic White	1 [Reference]	NA
Other^b^	1.46 (1.22-1.74)	<.001
Sex		
Male	1 [Reference]	NA
Female	0.86 (0.80-0.93)	<.001
Medicare and/or Medicaid enrollment status		
Medicare only	1 [Reference]	NA
Dual Medicare and Medicaid	1.05 (0.96-1.15)	.32
Residence		
Urban or suburban	1 [Reference]	NA
Rural	1.05 (0.93-1.18)	.45
Residence prior to hospice admission		
Community	1 [Reference]	NA
Nursing home	0.47 (0.40-0.54)	<.001
**Patient health characteristics**		
Frailty Index		
Not frail	1 [Reference]	NA
Prefrail	1.15 (0.99-1.34)	.08
Mildly frail	1.19 (1.02-1.38)	.03
Moderately to severely frail	1.15 (0.96-1.37)	.13
CMS HCC score	1.09 (1.07-1.12)	<.001
Principal diagnosis		
ADRD	0.92 (0.83-1.01)	.08
Cancer	1.45 (1.29-1.62	<.001
Cardiovascular disease	1.14 (1.03-1.27)	.01
Chronic kidney disease	1.17 (0.98-1.40)	.09
COPD	1.30 (1.13-1.49)	<.001
Other	1 [Reference]	NA
**Health care provision**		
Place hospice was received		
Community	1 [Reference]	NA
Assisted living	0.67 (0.59-0.77)	<.001
Nursing home	1.16 (1.02-1.33)	.03
Inpatient hospital	1.49 (1.12-1.98)	.007
Hospice residence	0.63 (0.45-0.88)	.007
Other	0.48 (0.32-0.73)	.001
Type of hospice care		
Continuous home care	1.27 (0.96-1.67)	.09
Inpatient respite	0.85 (0.69-1.04)	.11
General inpatient care	1.01 (0.85-1.20)	.94
Length of stay, d		
≤7	1.71 (1.53-1.90)	<.001
8-179	1 [Reference]	NA
≥180	0.60 (0.52-0.69)	<.001
Discharge reason		
Condition stabilization	1 [Reference]	NA
With cause	2.12 (1.51-2.99)	<.001
Patient unavailability	4.98 (4.00-6.19)	<.001
Revocation	3.21 (2.80-3.67)	<.001
Transfer to inpatient care	9.59 (7.93-11.60)	<.001
Transfer to other facilities	1.79 (0.90-3.54)	.08
ACP before hospice discharge	0.99 (0.87-1.12)	.86
Palliative care in 6 mo before hospice admission	1.27 (1.15-1.39)	<.001
**Organizational setting characteristics**		
Hospice size, quintile		
1 (Smallest)	1 [Reference]	NA
2	0.95 (0.79-1.15)	.62
3	1.01 (0.84-1.20)	.98
4	1.11 (0.93-1.32)	.23
5 (Largest)	0.94 (0.79-1.11)	.45
Hospice ownership status		
Not-for-profit	1 [Reference]	NA
For-profit	1.32 (1.15-1.52)	<.001
Government	1.38 (1.01-1.87)	.04
Other	1.17 (0.98-1.38)	.08

Abbreviations: ACP, advance care planning; ADRD, Alzheimer disease and related dementias; aOR, adjusted odds ratios; CMS, Centers for Medicare & Medicaid Services; COPD, chronic obstructive pulmonary disease; HCC Score, Hierarchical Condition Category; NA, not applicable.

^a^ Type 2 transitions: hospitalization within two days of hospice live discharge followed by in-hospital death. Analysis also adjusts for hospice admission and discharge years.

^b^ Includes American Indian or Alaska Native, Asian or Pacific Islander, or unknown.

### Estimated Probabilities

Black individuals had a 2.8–percentage point higher probability of experiencing a type 1 transition and a 1.2–percentage-point higher probability of experiencing a type 2 transition, compared with non-Hispanic White individuals (Table 4). Compared with care at nonprofit hospices, care at a for-profit hospice was associated with 3.5–percentage point higher probability of experiencing a type 1 transition and a 0.5–percentage-point higher probability of experiencing a type 2 transition. Palliative care consultations were associated with a 0.5–percentage point higher probability of type 2 transition. For experiencing type 1 transitions, any type of frailty was associated with 1.6– to 3.3–percentage point higher probability than nonfrailty. Receiving respite was associated with a 0.6–percentage point higher probability of experiencing a type 1 transition, and receiving GIP was associated with 0.9–percentage point lower probability of a type 1 transition (Table 4).

**Table 4.** Estimated Probabilities of Experiencing a Type 1 or Type 2 Burdensome Transition for Key Individual, Care-Related, and Systemic Factors

Characteristic	Percentage points (95% CI)	
	Type 1^a^	Type 2^b^
Patient race and ethnicity		
Hispanic	6.8 (5.9-7.6)	2.1 (1.8-2.5)
Non-Hispanic Black	9.2 (8.5-9.8)	2.9 (2.6-3.2)
Non-Hispanic White^c^	6.4 (6.1-6.7)	1.7 (1.6-1.8)
Other^d^	5.7 (5.0-6.5)	2.5 (2.0-3.0)
Patient Frailty Index score		
Not frail^c^	4.7 (4.2-5.1)	1.6 (1.4-1.8)
Prefrail	6.3 (6.0-6.6)	1.8 (1.7-2.0)
Mildly frail	6.8 (6.5-7.2)	1.9 (1.7-2.0)
Moderately to severely frail	8.0 (7.5-8.6)	1.8 (1.6-2.0)
Place hospice was received		
Community^c^	7.0 (6.6-7.3)	1.9 (1.8-2.1)
Assisted living	6.9 (6.4-7.3)	1.3 (1.1-1.5)
Nursing home	6.1 (5.6-6.6)	2.2 (2.0-2.5)
Inpatient hospital	5.3 (4.3-6.3)	2.8 (2.0-3.7)
Hospice residence	4.9 (3.8-5.9)	1.2 (0.8-1.6)
Other	3.7 (2.8-4.6)	0.9 (0.6-1.3)
Continuous home care		
Yes	9.4 (7.7-11.1)	2.3 (1.7-2.9)
No^c^	6.6 (6.3-6.9)	1.8 (17-2.0)
Inpatient respite		
Yes	5.3 (4.7-5.9)	1.6 (1.3-1.9)
No^c^	6.7 (6.4-7.0)	1.9 (1.7-2.0)
General inpatient care		
Yes	5.8 (5.0-6.5)	1.9 (1.5-2.2)
No^c^	6.7 (6.4-7.0)	1.8 (1.7-2.0)
Palliative care in 6 mo before hospice admission		
Yes	7.0 (6.5-7.5)	2.2 (2.0-2.5)
No	6.6 (6.3-6.9)	1.8 (1.7-1.9)
Ownership status		
Not-for-profit^c^	4.8 (4.4-5.2)	1.6 (1.4-1.8)
For-profit	8.3 (7.9-8.7)	2.1 (1.9-2.2)
Government	5.1 (3.8-6.4)	2.1 (1.5-2.7)
Other	6.2 (5.4-6.9)	1.8 (1.5-2.1)

^a^ Type 1 transitions: hospitalization within 2 days of hospice live discharge followed by hospice readmission within 2 days of hospital discharge.

^b^ Type 2 transitions: hospitalization within 2 days of hospice live discharge followed by in-hospital death.

^c^ Reference group.

^d^ Includes American Indian or Alaska Native, Asian or Pacific Islander, or unknown.

## Discussion

In this cohort study of Medicare FFS beneficiaries who were discharged alive from hospice, we examined factors associated with burdensome transitions 2 days after live hospice discharge. Live discharge from hospice is burdensome for individuals who are seriously ill and their families.^2,36^ Among the 15% of hospice patients discharged alive, 1 in 7 are either hospitalized within 2 days after discharge and readmitted to hospice or are hospitalized within 2 days after discharge and die while hospitalized. These transitions may signal problems with assessments of patient stability prior to discharge,^6^ lack of systematic approaches to live discharge planning, or disincentives for hospices to provide certain types of costly care, such as GIP.

Some patient sociodemographic characteristics were associated with protection against experiencing a burdensome transition after live discharge. Older age and female sex were associated with lower odds of experiencing either burdensome transition, while dual Medicare and Medicaid enrollment was associated with protection against type 1 transitions only. Medicaid-enrolled individuals may be eligible for additional home- and community-based services, such as the 1915(c) waiver,^37^ that were associated with protection against hospitalization and hospice readmission after hospice discharge. Residing in a nursing home prior to hospice admission was also associated with protection against burdensome transitions. Nursing homes are more familiar with end-of-life trajectories and may make more appropriate referrals to hospice. Patients remaining in the same care setting with staff familiar with their evolving care needs may benefit from this continuity and face fewer burdensome transitions after hospice discharge.

In contrast, identifying as Black was associated with higher odds of either type of burdensome transition; identifying as Hispanic was associated with higher odds of type 2 burdensome transition. Our findings identify another layer of hospice-related disparity and risk for individuals from racially and ethnically minoritized groups: Black and Hispanic individuals access hospice at lower rates than non-Hispanic White individuals,^34^ experience live discharge at higher rates,^9,10^ and are also at increased risk of burdensome transitions after live discharge. Consistent with lower rates of hospice enrollment, Hispanic individuals may be less likely to reenroll in hospice, including if they are hospitalized, which may explain the lack of association between Hispanic identity and type 1 burdensome transitions. Structural factors, such as inequitable distribution of and access to health care resources and institutionalized racism, are important contributing factors in observed racial and ethnic disparities in health outcomes.^38,39^ In addition to addressing structural inequities, careful attention to the needs of individuals at increased risk for burdensome postdischarge transitions may help prevent them from occurring.^18^

Factors related to health care provision were associated with burdensome transitions after live discharge. These are potentially modifiable, making them promising intervention targets. Longer hospice stays were associated with lower odds of burdensome transitions. Although discouraged by regulations,^40,41^ longer stays may allow the hospice team to stabilize individuals who are seriously ill and establish care plans, which may be beneficial after hospice services cease. Inpatient respite and GIP were associated with lower odds of hospitalization and hospice readmission but not hospitalization and hospital death. These types of hospice care represent only 6.2% of hospice spending^34^ due to restrictive eligibility criteria and limited availability. Our findings suggest they may be effective in supporting patients with complicated needs requiring temporary hospitalization. Increasing availability of inpatient respite and GIP within the hospice benefit may reduce burdensome transitions after live discharge. The lack of association between type of hospice care and type 2 transitions may relate to insufficient power to detect associations, as type 2 transitions, inpatient respite, and GIP occurred infrequently in our sample. Individuals receiving hospice in assisted living or a hospice residence had lower odds of hospitalization and hospital death but not hospitalization and hospice readmission. There may be support structures and professional medical care in these settings that prevent individuals from being hospitalized and dying in hospital after live discharge. Shorter hospice stays were associated with higher odds of burdensome transitions. Shorter stays likely reflect late referrals and do not allow the hospice team to put an effective care plan in place, potentially leading to additional transitions if live discharge occurs.

Although we could not assess the ongoing nature of goals-of-care planning, having a palliative care consultation in the months leading up to hospice admission was associated with higher odds of burdensome transitions. We would expect that palliative care would facilitate a timely transition into hospice^42^ and be associated with lower likelihood of hospital death.^43^ However, we found that palliative care encounters were associated with higher odds of burdensome transitions after live discharge. Possibly, palliative care consultations are sought for complex patients for whom hospice provides stability, but complications reoccur following live discharge, increasing risk for burdensome transition.

At the organizational level, individuals who received care from for-profit hospices had higher odds of a burdensome transition, possibly signaling a reverberating impact of poorer quality care documented in for-profit hospice agencies.^16,25,44^ Financial incentives to discharge patients alive to reduce costs^25^ may also contribute to postdischarge burdensome transitions. Hospices may discharge patients who require hospitalization and readmit them on hospital discharge to avoid paying for costly hospice care, such as GIP. Individuals receiving care at large hospices had higher odds of experiencing a hospitalization and hospice readmission. Larger hospice agencies may enroll patients with more complicated needs who require hospitalization for complex symptom management.

### Limitations

Our study has limitations. First, our results are only applicable to individuals receiving FFS Medicare and may not be generalizable to Medicare Advantage enrollees. However, Medicare Advantage did not have a hospice benefit during the study period, and prior studies have found lower rates of live discharge in Medicare Advantage populations compared with Medicare FFS.^45^ Second, type 2 burdensome transitions (hospitalization followed by hospital death) were relatively uncommon: only 3000 individuals experienced this type of transition in a 6-year period, and so differences may not be detected for this group. Although hospitalization during a longer period after live discharge may be more common, we aligned our analysis with the CMS definition, given the policy relevance. Moreover, hospital admission within 2 days of live discharge is highly disruptive for patients and families and therefore important to consider. Third, we are unable to capture process-related measures, key in understanding and addressing adverse health outcomes. We used proxy measures to represent these processes (eg, advance care planning, palliative care consultations). Fourth, other factors not captured in claims data, such as family burden and resources and availability of paid and unpaid caregivers, may be protective against burdensome transitions. We have attempted to address potential bias by examining a comprehensive set of factors that may explain burdensome transitions.

## Conclusions

This cohort study found that burdensome transitions following live discharge from hospice were associated with patient, health care provision, and organizational setting characteristics that require responses in clinical practice, policy, and research. In clinical practice, increased attention to the needs of persons from racially and ethnically minoritized groups and with more complicated care needs and frailty is required, as these patients may be susceptible to postdischarge burdensome transitions. Introduction of systemic discharge planning—in clinical practice and supported by policy—may alleviate burdensome transitions in individuals discharged alive from large, for-profit hospice agencies and receiving hospice in nursing homes. Policy facilitating patient access to and hospice-hospital partnership for GIP and inpatient respite services and adjusting hospice payment structures to disincentivize discharge among individuals needing this type of intensive care may reduce postdischarge hospitalization and readmission. Additional research is needed to understand the association between palliative care consultations and burdensome transitions after live hospice discharge, whether these consultations are a marker for patients with particularly complex needs that continue until death, and whether they may have an unintended negative long-term effect on individuals discharged alive from hospice.

## Notes

Supplement.
Data Sharing Statement
