# Supplementary material for: Hospice Readmission, Hospitalization, and Hospital Death Among Patients Discharged Alive from Hospice
Source: JAMA Netw Open. 2024 May 16;7(5):e2411520. doi: 10.1001/jamanetworkopen.2024.11520 (PMC11099680; doi:10.1001/jamanetworkopen.2024.11520)
Supplement: Supplement. — Data Sharing Statement [file jamanetwopen-e2411520-s001.pdf]

Data Sharing Statement

Luth. Hospice Readmission, Hospitalization, and Hospital Death Among Patients Discharged Alive from Hospice. *JAMA Netw Open*. Published May 16, 2024.
doi:10.1001/jamanetworkopen.2024.11520

Data
Data available: No

Additional Information
Explanation for why data not available: Per CMS data use agreements, data cannot be shared.
